# A new species of *Denisiphantes* Tu, Li & Rollard, 2005 (Araneae, Linyphiidae) from Yunnan, China

**DOI:** 10.3897/zookeys.1023.62025

**Published:** 2021-03-10

**Authors:** Guchun Zhou, Muhammad Irfan, Xianjin Peng

**Affiliations:** 1 1College of life Sciences, National Navel Orange Engineering Research Center, Gannan Normal University, Ganzhou 341000, Jiangxi, China Gannan Normal University Ganzhou China; 2 College of Life Sciences, Hunan Normal University, Changsha, Hunan Province, 410081, China Hunan Normal University Changsha China; 3 Key Laboratory of Eco-environments in Three Gorges Reservoir Region (Ministry of Education), School of Life Sciences, Southwest University, Chongqing 400715, China Southwest University Chongqing China

**Keywords:** Copulatory organ, Micronetinae, Southeast Asia, sheet-web spiders, taxonomy

## Abstract

*Denisiphantes arcuatus***sp. nov.** (♂♀) is described from Yunnan, China. Detailed descriptions of somatic features, genitalic characters, photos of habitus of the new species as well as of copulatory organs of *Denisiphantes denisi* (Schenkel, 1963) (♂♀) are presented. A distribution map is also provided.

## Introduction

**Figure 1.**
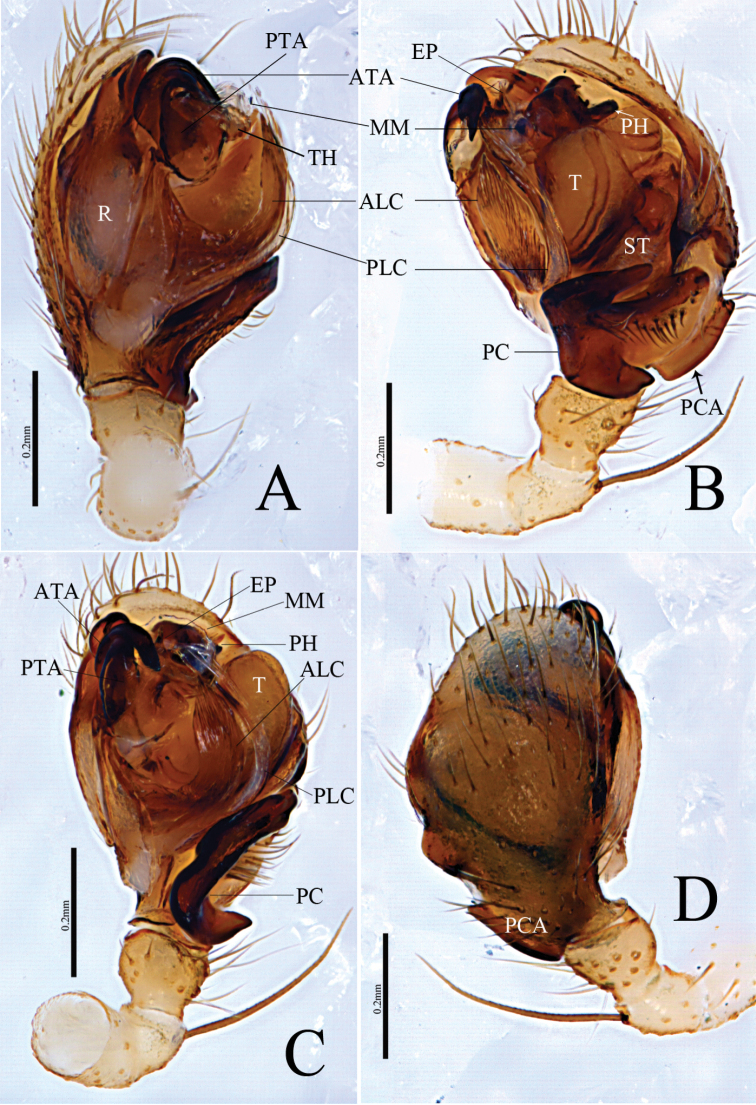
*Denisiphantes arcuatus* sp. nov., male holotype palp **A** prolateral view **B** retrolateral view **C** ventral view **D** dorsal view.

**Figure 2.**
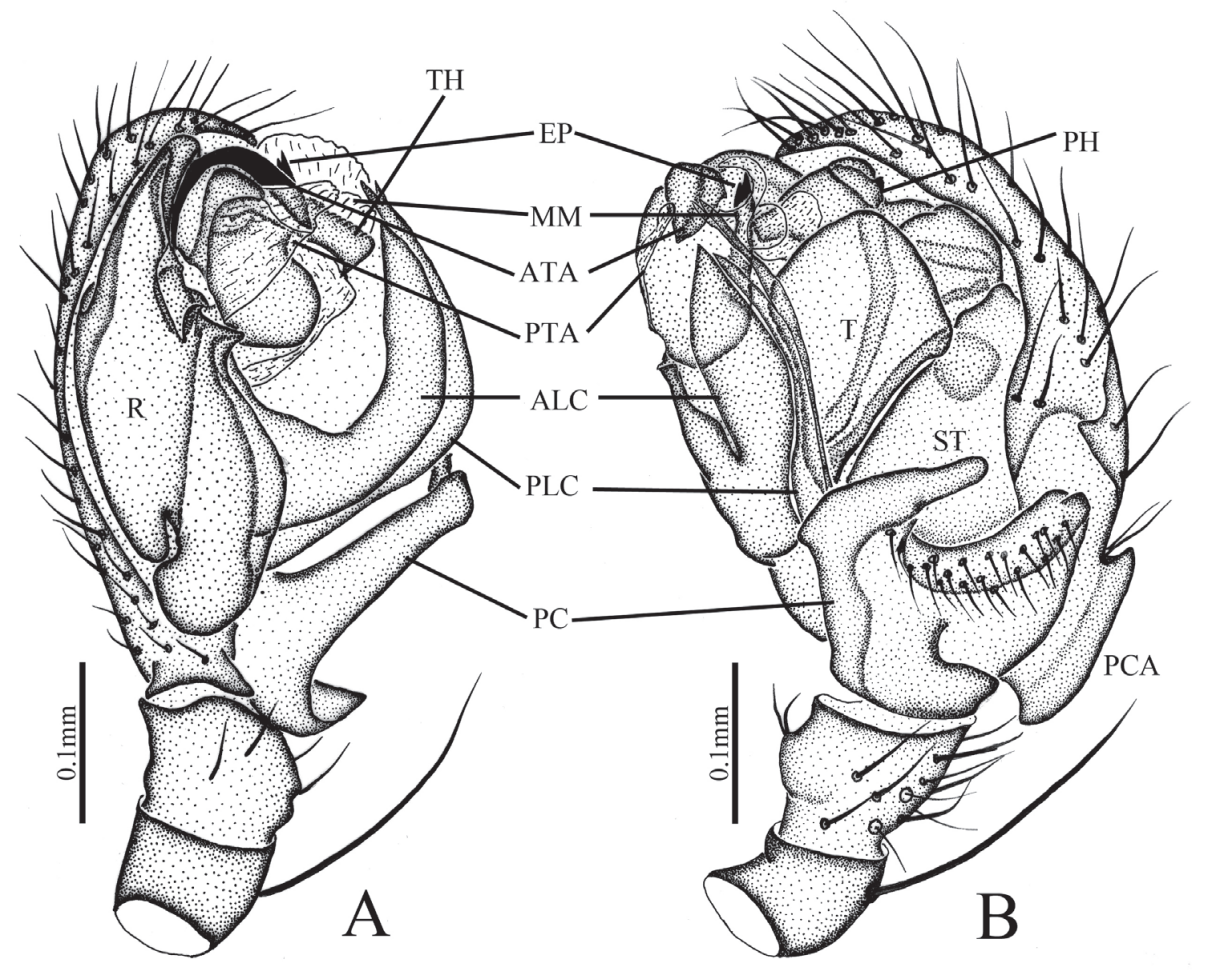
*Denisiphantes arcuatus* sp. nov., male holotype palp. **A** prolateral view **B** retrolateral view.

**Figure 3.**
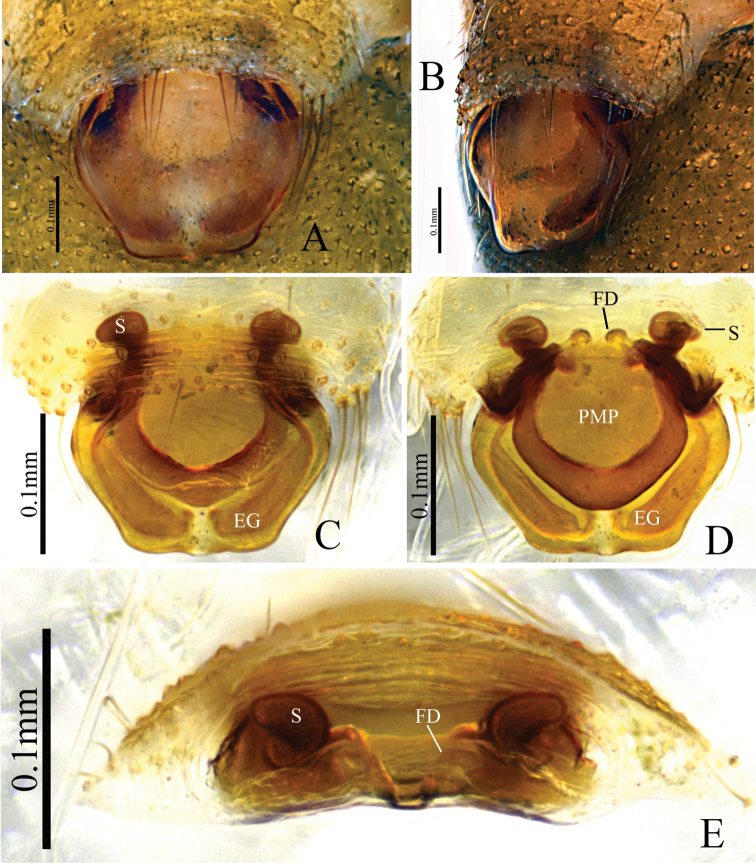
*Denisiphantes arcuatus* sp. nov., female paratype **A, C** epigynum, ventral view **B** epigynum, lateral view **D** vulva, dorsal view **E** vulva, anterior view.

**Figure 4.**
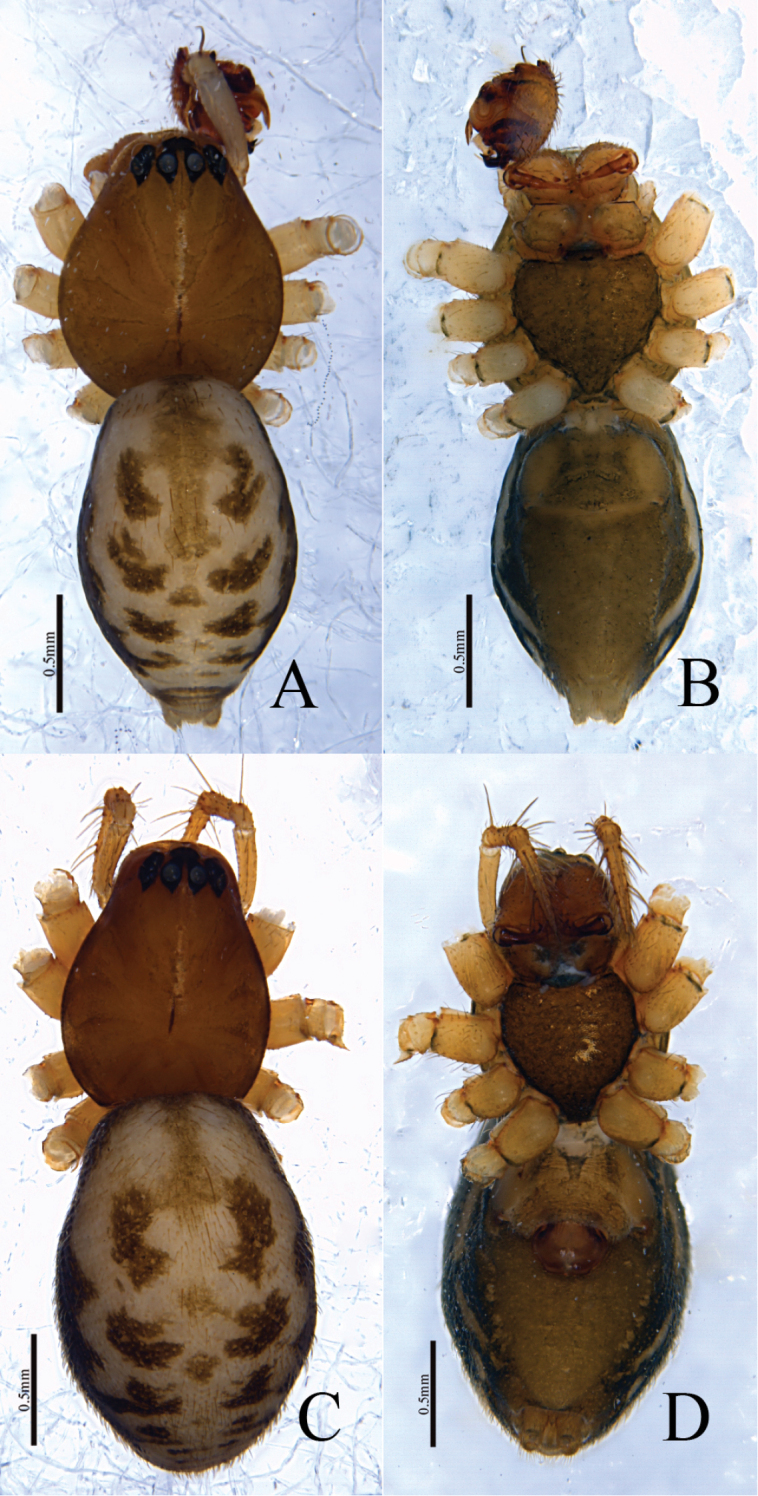
*Denisiphantes arcuatus* sp. nov., male holotype (**A, B**) and female paratype (**C–D**) **A, C** habitus, dorsal view **B, D** habitus, ventral view.

**Figure 5.**
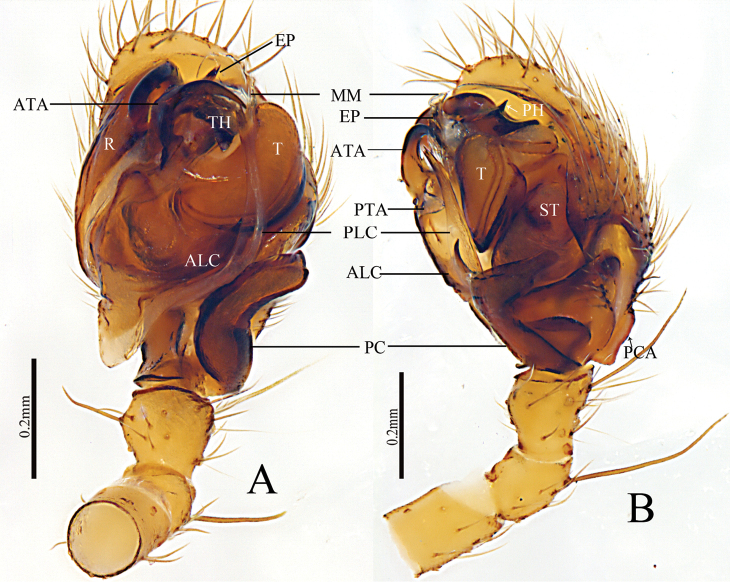
*Denisiphantes denisi* (Schenkel, 1963) **A** palp, ventral view **B** palp, retrolateral view.

**Figure 6.**
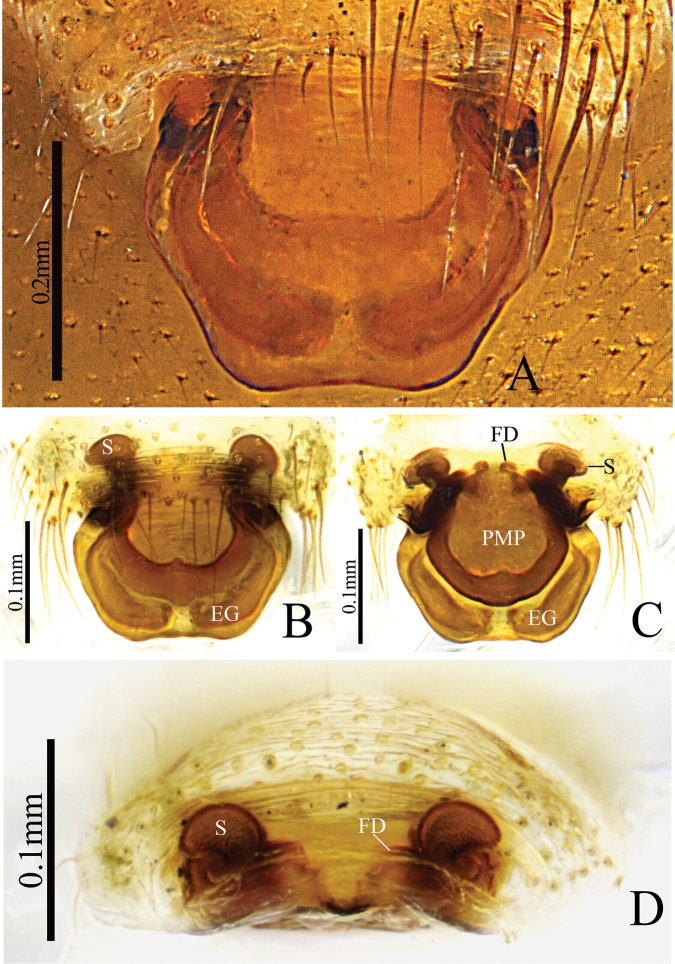
*Denisiphantes denisi* (Schenkel, 1963) **A, B** epigynum, ventral view **C** vulva, dorsal view **D** vulva, anterior view.

**Figure 7.**
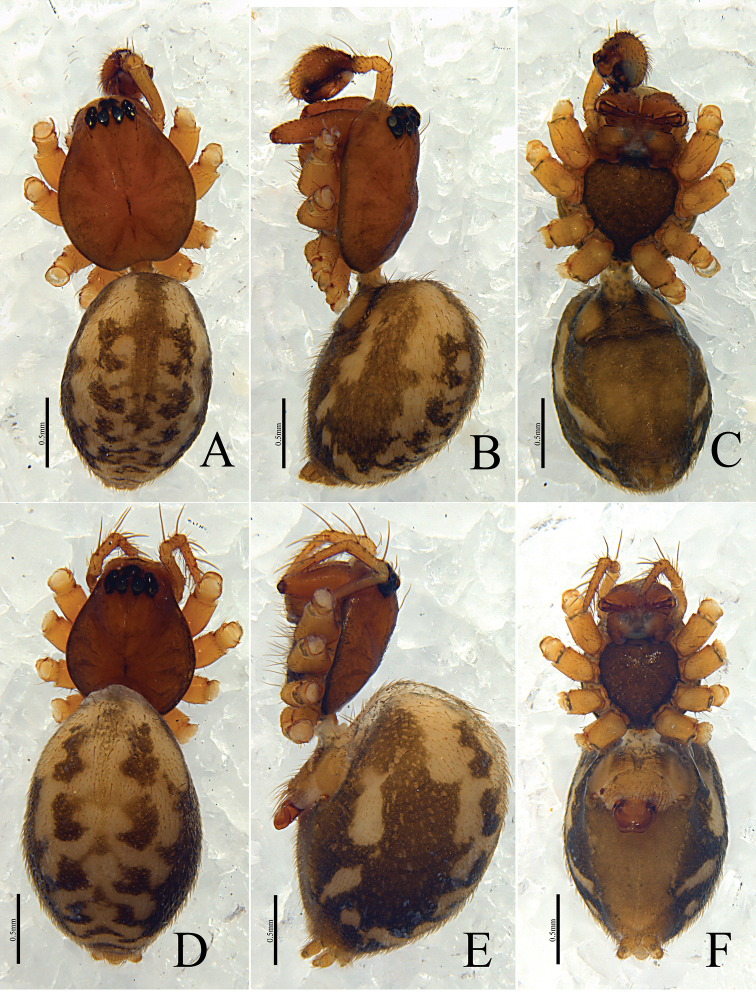
*Denisiphantes denisi* (Schenkel, 1963), male (**A–C**) and female (**D–F**) **A, D** habitus, dorsal view **B, E** habitus, lateral view **C, F** habitus, ventral view.

**Figure 8.**
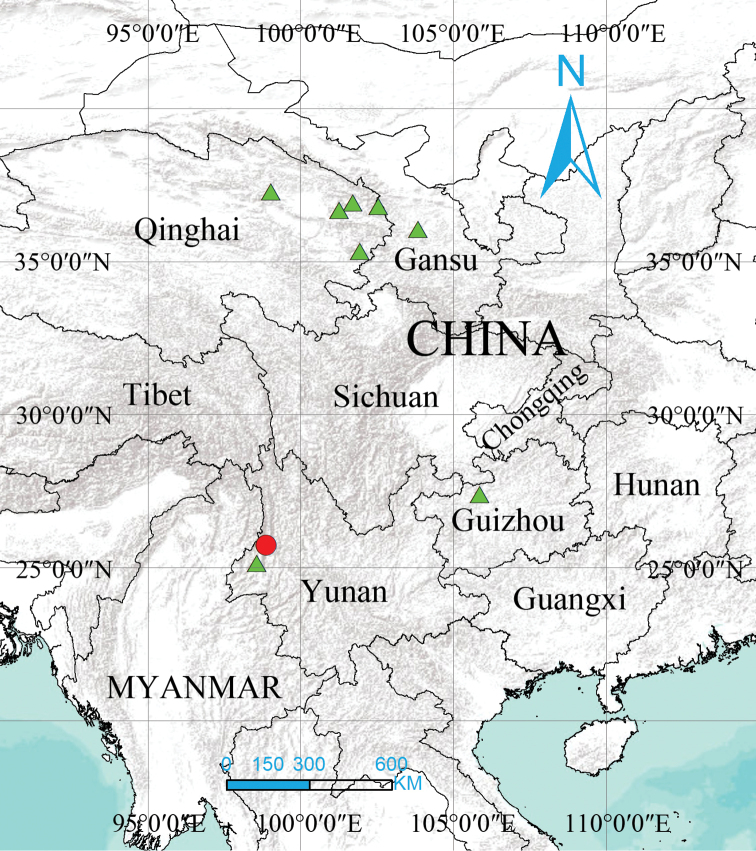
Distribution of *Denisiphantes arcuatus* sp. nov. and *Denisiphantes denisi* (Schenkel, 1963).

Li (2020) listed 403 Chinese linyphiid species from 162 genera, of which 120 species from 44 genera belong to Micronetinae (Li and Lin 2016). Spider collections made in the Yunnan Province between 2002–2006 revealed a new Linyphiidae species herein described. The new species belong in the monotypic genus, *Denisiphantes* Tu, Li & Rollard, 2005. The type species, *Denisiphantes denisi* (Schenkel, 1963), was described based on a female specimen from Ganzu Province and it also occurs in Qinghai Province (Hu 2001; Tu, Li and Rollard 2005). The male was later described by Zhu and Li (1983). Color photos of both species are presented (Figs 1–7) as well as a detailed morphological description, a diagnosis, and a locality map (Fig. 8).

## Material and methods

Specimens were collected by hand collecting and beating shrubs and were kept in 75% ethanol. After dissection, the epigyna were cleared in trypsin enzyme solution before examination and photography. The left male palps were used for description and illustration. Specimens were examined and measured with a Leica M205C stereomicroscope. Photos were taken with a digital Leica MC170 HD camera mounted on a Leica M205C and were stacked by Helicon Focus software (3.10.). The map (Fig. 8) was created using ArcMap 10.2, and then modified using Adobe Photoshop CS2 Extended. Leg measurements are given in the following order: total length (femur, patella + tibia, metatarsus, tarsus). All measurements are given in millimeters (mm). The terminology used in text and figure legends follows Tu et al. (2005). The type specimens are deposited at the College of Life Sciences, Hunan Normal Universit (**HNU**), Changsha, China.

### Abbreviations

**AER** anterior eye row

**ALC** anterior projection of lamella characteristica

**ALE** anterior lateral eye

**AME** anterior median eye

**AME–ALE** the distance between AME and ALE

**AME–AME** the distance between AMEs

**ATA** anterior terminal apophysis

**EG** entrance groove

**EP** embolus proper

**MM** median membrane

**PC** paracymbium

**PCA** proximal cymbial apophysis

**PER** posterior eye row

**PH** pit hook on suprategulum

**PLE** posterior lateral eye

**PLC** posterior projection of lamella characteristica

**PME** posterior median eye

**PME–PLE** distance between PME and PLE

**PME–PME** distance between PMEs

**PMP** posterior median plate

**PTA** posterior terminal apophysis

**R** radix

**S** spermatheca

**ST** subtegulum

**T** tegulum

**TH** thumb of embolus

## Taxonomy

### Order Araneae Clerck, 1757

#### Family Linyphiidae Blackwall, 1859

**Subfamily Micronetinae Hull, 1920**

**Genus *Denisiphantes* Tu, Li & Rollard, 2005**

##### Denisiphantes arcuatus sp. nov.

Taxon classificationAnimaliaAraneaeLinyphiidae

CDDA83B8-CF1B-5523-AC9D-59CA1EA4EA4D

http://zoobank.org/E5E1CCE9-291E-4137-9B74-26EF67FB9308

Figures 1 , 2 , 3 , 4

###### Material examined.

***Holotype***: male, China, Yunnan Province, Nujiang Prefecture, Sanjiang Township, Lushui County, Nu Jiang, 25.72964°N, 98.87180°E, alt. 790 m, 26.VI.2000, leg. D. H. Kavanaugh, Charles Griswold & Heng–mei Yan (HNU–00–Lan–1). ***Paratypes***: 2 females, collected together with the holotype (HNU–00–Lan–2~3).

###### Diagnosis.

This new species resembles *Denisiphantes denisi* (Schenkel, 1963) in having a similar long ridge-shaped proximal cymbial apophysis of the cymbium in the male palp and a broad scape, almost hexagonal in shape in the epigynum, but can be distinguished by the following characters: (1) Lower margin of distal arm of the paracymbium with a projection, wider than long with a blunt end and covering the posterior margin of the tibia in retrolateral view in the new species (Figs 1A–D, 2A, B), whereas the projection of the lower margin of the distal arm in *D. deni* is longer than wide with a pointed end and not covering the posterior margin of the tibia (Fig. 5A, B); (2) Anterior projection of the lamella characteristica (ALC) leaf-shaped with pointed end, protruding above the tegulum and the posterior projection of the lamella characteristica (PLC) as long as the ALC with pointed end (Figs 1A–D, 2A, B), whereas ALC short, almost equal to the suprategulum (ST) with pointed end and PLC long, almost touching the anterior terminal apophysis (ATA) in *D. denisi* (Fig. 5A, B); (3) Anterior terminal apophysis (ATA) strongly curved with blunt end and pointing towards ALC in new species (Figs 1A–D, 2A, B), whereas slightly curved with pointed end and pointing towards the tegulum in ventral view in *D. denisi* (Fig. 5A, B); (4) Posterior terminal apophysis (PTA) triangular in the new species (Figs 1A, 2A), whereas it is tongue-shaped in *D. denisi* (Tu et al. 2005, figs 16, 17; Fig. 5A, B). (5) Female posterior median plate (PMP) tetragonal shape in the new species (Fig. 3D), whereas it is hexagonal in *D. denisi* (Fig. 6C).

###### Description.

**Male** (holotype, HNU–00–Lan–1): Total length: 2.57. Carapace 1.06 long, 0.97 wide, cephalic region slightly elevated, brown, fovea, cervical and radial grooves distinct. Clypeus 0.35 high. Sternum wider than long, brown, with spine-like setae; labium wider than long; maxillae long, distal-end broad with scopulae. Chelicerae with 5 retromarginal teeth, promarginal teeth absent. Eye region narrow, AER recurved, PER straight, slightly wider than AER. Eye sizes and interdistances: AME 0.06, ALE 0.08, PME 0.09, PLE 0.08, AME–AME 0.03, PME–PME 0.05, AME–ALE, 0.08, PME–PLE 0.07, AME–PME 0.06, ALE–ALE 0.40, PLE–PLE 0.43, ALE–PLE contiguous. Length of legs: I 5.89 (1.48, 1.91, 1.53, 0.97), II 5.03 (1.31, 1.57, 1.31, 0.84), III 3.9 (1.12, 1.19, 0.97, 0.62), IV 5.32 (1.49, 1.61, 1.38, 0.84). Leg formula (longest to shortest legs): I–IV–II–III. TmI 0.66 and TmIV 0.48. Tibial spine formula: 2–2–2–2. Abdomen 1.51 long, 0.94 wide, oval, light grey, with distinct pattern on dorsal surface from proximal end to base of spinnerets and extending laterally, ventral side green. Palp (Figs 1A–D, 2A, B): patella short, with long dorsal spine; tibia conic, with two retrolateral and one dorsal trichobothria; paracymbium U–shaped, basal part with several setae, lower margin of distal arm with a projection, wider than long with a blunt end, covering the posterior margin of tibia in retrolateral view; cymbium rather long, ridge-shaped, proximal to cymbial apophysis (PCA). Pit hook curved with a pointed end. Embolic division: Radix long, sclerotized; anterior projection of lamella characteristica (ALC) leaf-shaped with a pointed end, protruding above tegulum; posterior projection of lamella characteristica (PLC) as long as ALC with a pointed end; terminal apophysis sclerotized, dark, strongly curved with a blunt end, pointing towards anterior projection of lamella characteristica (ALC). Embolus short, tip bifurcated; apical margin of thumb (TH) serrated, median membrane simple.

**Female** (paratype HNU–00–Lan–2): Total length: 3.16. Carapace 1.26 long, 0.92 wide; cephalic region slightly elevated, dark brown; fovea, cervical and radial grooves distinct. Clypeus 0.31 high. Sternum wider than long, brown, with spine-like setae; labium wider than long; maxillae long, distal-end broad with scopulae. Chelicerae with 4 promarginal and 3 retromarginal teeth. Eye region narrow, AER recurved, PER straight, slightly wider than AER. Eye sizes and interdistances: AME 0.07, ALE 0.09, PME 0.08, PLE 0.08, AME–AME 0.02, PME–PME 0.05, AME–ALE, 0.06, PME–PLE 0.06, AME–PME 0.06, ALE–ALE 0.39, PLE–PLE 0.41, ALE–PLE contiguous. Length of legs: I 5.55 (1.49, 1.82, 1.39, 0.85), II 4.8 (1.32, 1.55, 1.21, 0.72), III 3.83 (1.09, 1.27, 0.92, 0.55), IV 5.18 (1.46, 1.63, 1.33, 0.76). Leg formula I–IV–II–III. TmI 0.58 and TmIV 0.43. Tibial spine formula: 2–2–2–2. Abdomen 1.90 long, 1.16 wide; oval, light grey, dorsally with distinct pattern from proximal end to base of spinnerets and extending laterally, ventral side green. Epigynum (Fig. 3A–E): broad, posteriorly pointing scape almost hexagonal-shaped, stretcher and lateral pockets absent; posterior median plate (PMP) conspicuously large, tetragonal-shaped, covering most of dorsal side of scape; spermathecae elliptical.

###### Etymology.

The species name comes from the Latin adjective “*arcuatus*” meaning “curved” and refers to the curved anterior terminal apophysis in the male palp.

###### Distribution.

Known only from the type locality in Yunnan, China (Fig. 8).

##### Denisiphantes denisi

Taxon classificationAnimaliaAraneaeLinyphiidae

(Schenkel, 1963)

55B818CA-1833-58D4-BB15-DB3539EC7DD8

Figures 5 , 6 , 7

Lepthyphantes denisi Schenkel, 1963: 118, fig. 70a–c.
Lepthyphantes denisi Zhu & Li, 1983: 146, fig. 3d–f.
Lepthyphantes denisi Hu, 2001: 503, fig. 334.1–4.
Denisiphantes denisi Tu, Li & Rollard, 2005: 652, figs 11–25.
Denisiphantes denisi Tanasevitch, 2006: 303, figs 76, 77.

###### Material examined.

3♂3♀, China, **Yunnan Province**, Tengchong County, Beihai Township, 15.2 km NE of Tengchong at Qinghai (lake), 25.13408°N, 98.57144°E, alt. 1842 m, 07 June 2006, D. H. Kavanaugh, R. L. Brett & Da–zhi Dong (HNU–DHK–2006–060); 1♀, **Guizhou Province**, Dafang County, Xingshu Township, Cuisuba, 27°23'N, 105°52'E, alt. 1750 m, 15 August 2020, Zhang Mao (HNU–202008–W–5–10); 1♀, Dafang County, Xingshu Township, Cuisuba, 27°23'N, 105°52'E, alt. 1750 m, 17 August 2020, Zhang Mao (HNU–202008–S–3–20).

###### Distribution.

China (Gansu, Guizhou, Qinghai and Yunnan, Fig. 8).

## Discussion

*Denisiphantes* was described as a monotypic genus based on the material collected from Qinghai and also reported from Gansu and Guizhou. The new species described here is reported from Yunnan. Considering the vast distribution of the type species (Fig. 8), it is possible that there are still several species of this genus that need to be explored from Yunnan and across the adjacent areas to understand the distribution and origin of this genus.

## Supplementary Material

XML Treatment for Denisiphantes arcuatus

XML Treatment for Denisiphantes denisi
